# Extracellular DNA and Deoxyribonuclease Activity as Prognostic Markers in Sepsis

**DOI:** 10.3390/biomedicines12112565

**Published:** 2024-11-09

**Authors:** Monika Janíková, Nikola Pribulová, Katarína Kmeťová, Kristína Macáková, Anna Dobišová, Michaela Kopčová, Mária Bucová, Barbora Vlková, Peter Celec

**Affiliations:** 1 Institute of Molecular Biomedicine, Faculty of Medicine, Comenius University, Sasinkova 4, 811 08 Bratislava, Slovakia; monika.janikova@fmed.uniba.sk (M.J.); pribulova.n@gmail.com (N.P.); kmetova.kat@gmail.com (K.K.); kristina.macakova@fmed.uniba.sk (K.M.); barbora.vlkova@fmed.uniba.sk (B.V.); 2 1st Department of Anesthesiology and Intensive Care Medicine, Faculty of Medicine, Comenius University and University Hospital, 826 06 Bratislava, Slovakia; anna.dobisova@fmed.uniba.sk; 3 Institute of Immunology, Faculty of Medicine, Comenius University, 811 08 Bratislava, Slovakia; michaela.kopcova@fmed.uniba.sk (M.K.); maria.bucova@fmed.uniba.sk (M.B.); 4 Institute of Pathophysiology, Faculty of Medicine, Comenius University, 811 08 Bratislava, Slovakia

**Keywords:** immune dysregulation, cell-free DNA, biomarker, nucleases, circulating nucleic acids

## Abstract

**Background/Objectives**: Sepsis is characterized by a dysregulated immune response to infection and is associated with high lethality. Extracellular DNA (ecDNA) has drawn significant interest as a damage-associated molecular pattern because of its potential involvement in the pathophysiology of sepsis. **Methods**: In this study, we examined the ecDNA concentration in 27 adult patients admitted to the intensive care unit. Fluorometry and quantitative PCR were used for the assessment of ecDNA. In addition, deoxyribonuclease activity was measured as a potential modulator of ecDNA. **Results:** Our findings reveal nearly 5-fold higher concentrations of ecDNA in non-survivors, suggesting its potential as a prognostic indicator for sepsis outcomes on day 7. Interestingly, the subcellular origin of ecDNA was similar between patients diagnosed with systemic inflammatory response syndrome, sepsis, and septic shock. Deoxyribonuclease activity, implicated in the cleavage of ecDNA, was comparable across all patient groups. **Conclusions:** To establish the prognostic value of ecDNA as a biomarker, further investigations within a larger patient cohort are needed. Nevertheless, our results suggest that high ecDNA in sepsis patients represents a negative prognostic biomarker.

## 1. Introduction

While the definition of sepsis is continuously evolving, it represents a state of life-threatening organ dysfunction caused by a dysregulated host response to infection [1,2]. The mortality and the lethality of sepsis remain high, despite the advances in understanding its pathogenesis [3]. Besides difficulties in sepsis prevention, one of the key issues is the lack of early diagnostic and prognostic biomarkers that would reliably help to identify and stratify patients [4]. Biomarkers should ideally be specific, sensitive, and related to the pathophysiology of sepsis, but also valid for most if not all causes of sepsis.

Most DNA is located in the nucleus and mitochondria of eukaryotic cells. This prevents DNA from being recognized as a damage-associated molecular pattern that can activate an immune response [5]. Even in physiological situations, some DNA is outside of the cells—this extracellular DNA (ecDNA), often termed cell-free DNA, stems from cell turnover and might have a role in maintaining immune homeostasis [6]. Its composition can be analyzed by sequencing; this has revolutionized non-invasive prenatal diagnosis [7] and is changing the possibilities of cancer screening [8].

As sequencing does not provide information about the quantity of ecDNA, this is often ignored in studies analyzing ecDNA. The concentration of ecDNA in plasma can be increased by trauma [9], pre-eclampsia [10], and cancer [11], but in most diseases, the source of ecDNA is activated immune cells. Thus, the reason for an increase in ecDNA is often inflammation [12]. However, the association between inflammation and ecDNA is bi-directional.

Endogenous ecDNA is either of a nuclear (ncDNA) or mitochondrial (mtDNA) origin and has pro-inflammatory effects if not cleaved rapidly. EcDNA, especially mtDNA, activates the cells of the immune system, causing an inflammatory response [5]. The pro-inflammatory properties of ecDNA could be a key part of the pathomechanism of sepsis [13]. MtDNA shares similarities with bacterial DNA and can, thus, be recognized by pattern recognition receptors [14] with potential roles in the pathogenesis of sepsis [15].

Deoxyribonucleases (DNases) are intracellular or extracellular enzymes which hydrolyze the phosphodiester bond of DNA released from dying cells [16]. The most abundant endonuclease secreted into the blood stream to cleave ecDNA is DNase I [16]. Our previous experimental study showed that exogenous DNase decreases mortality in an animal model of sepsis [17]. Whether endogenous DNase variability affects progress and the survival of sepsis is unclear.

The aim of our study was to evaluate ecDNA as a prognostic marker in sepsis. We hypothesized that patients with worse clinical manifestations of sepsis will have higher concentrations of ecDNA in plasma and lower DNase activity, with slow cleavage potentially explaining the high ecDNA. When the subcellular origin of ecDNA was evaluated, we hypothesized that both ncDNA and mtDNA are higher in sepsis patients with worse prognoses.

## 2. Methods

### 2.1. Subjects and Sample Collection

Blood samples were collected from 27 adult patients (17 men and 10 women; listed in Table 1) admitted to the intensive care unit (ICU) of the 1st Department of Anaesthesiology and Intensive Care Medicine, Faculty of Medicine, Comenius University and University Hospital, Bratislava, Slovakia. All patients were examined by physicians and diagnosed based on valid guidelines and recommendations [18]. Inclusion criteria included patients who were suspected of or had ongoing systemic inflammation, sepsis, septic shock, or non-infectious systemic inflammation with the hospitalization length expected to be more than 24 h. Patients with an age below 18 years or a primary immunodeficiency disease, patients undergoing immunosuppressive therapy, and patients in the terminal stage of the disease, with death expected within 24 h of admission to the ICU, were excluded from this study. This study was approved by the Ethical Committee of the Faculty of Medicine, Comenius University in Bratislava and University Hospital, Bratislava, Slovakia, and informed consent was obtained from each patient or her/his legally authorized representative.

### 2.2. Extracellular DNA Isolation and Quantification

In this study, 3 mL of blood was collected into EDTA-coated tubes and 5 ml of blood into serum-separating tubes within 24 h of patient admission to the ICU. The patients were monitored and treated during hospitalization, but we analyzed only one sample per patient from the time point of admission to test the potential prognostic value of clinical applicability. Samples were centrifuged at 3000× *g* and 4 °C for 10 min. Collected plasma was centrifuged again at 16,000× *g* and 4 °C for 10 min. Subsequently, ecDNA was isolated from 200 µL of plasma using QIAamp DNA Blood Mini Kit (Qiagen, Hilden, Germany) according to the manufacturer’s instructions. The total ecDNA isolated from human plasma was quantified with a Qubit Fluorometer 3.0 using a Qubit dsDNA HS Assay Kit (Thermo Fisher Scientific, Waltham, MA, USA).

### 2.3. Real-Time PCR for Quantification of ncDNA and mtDNA

Real-time PCR was performed using primers designed for human ncDNA (β-globin: Fw 5′-GCTTCTGACACAACTGTGTTC-3′; Rv 5′-CACCAACTTCATCCACGTTCA-3′) and human mtDNA (D-loop: Fw 5′-CATAAAAACCCAATCCACATCA-3′; Rv 5′-GAGGGGTGGCTTTGGAGT-3′). All primers were synthesized by Microsynth AG (Balgach, Switzerland). The reaction mix was set up to be 10 µL and contained 5 µL of SsoAdvanced universal SYBR^®^ Green supermix (2×) (Bio-rad, Hercules, CA, USA), 250 nM of forward and reverse primers for β-globin or D-loop gene, 2.5 µL of DNA template, and 2 µL of molecular-grade water. Real-time PCR was performed on an Eppendorf realplex^4^ Mastercycler epgradient S (Eppendorf, Hamburg, Germany). Thermocycling conditions for β-globin gene were set to 98 °C for 3 min for initial denaturation, followed by 40 cycles of amplification: 98 °C for 15 s, 51° for 30 s, and 60 °C for 30 s. Thermocycling conditions for the D-loop gene are set at 98 °C for 3 min for initial denaturation, followed by 40 cycles of amplification: 98 °C for 15 s, 47° for 30 s, and 60 °C for 30 s. For all PCR, products a melting curve was obtained. PCR efficiency was between 90 and 110%.

### 2.4. Single Radial Enzyme Diffusion (SRED) for Measurement of DNase Activity

For DNase activity, 5 mL of blood was collected into serum-separating tubes within 24 h of patient admission to the ICU. After blood coagulation, samples were centrifuged at 1600× *g* and 4 °C for 10 min to obtain a serum. DNase activity was measured from human serum samples using 1% agarose gel containing 2 mM of MgCl_2,_ 2 mM of CaCl_2_, 20 mM of Tris-HCl, with pH = 7.5, and DNA from rat livers (0.35 mg/mL of gel). Serial dilutions of RNase-free DNase I (Qiagen, Hilden, Germany) were used for calibration curves. From each serum sample, 3 μL was pipetted into the gel and incubated overnight at 37 °C in the dark. After incubation, circle diameters were measured using ImageJ 1.54g software (NIH, Bethesda, MD, USA).

### 2.5. Statistical Analysis

Statistical analysis was performed using GraphPad Prism 10.1.2. Software, Inc., San Diego, CA, USA. Results were analyzed using a two-tailed t-test or one-way ANOVA or Pearson correlation test. P values of less than 0.05 were considered significant. Results are presented as means, with standard deviations given as error bars.

## 3. Results

### 3.1. Non-Survivors Have Higher Plasma ecDNA Concentrations by Day 7 Predicting Sepsis Outcome

**Figure 1:**
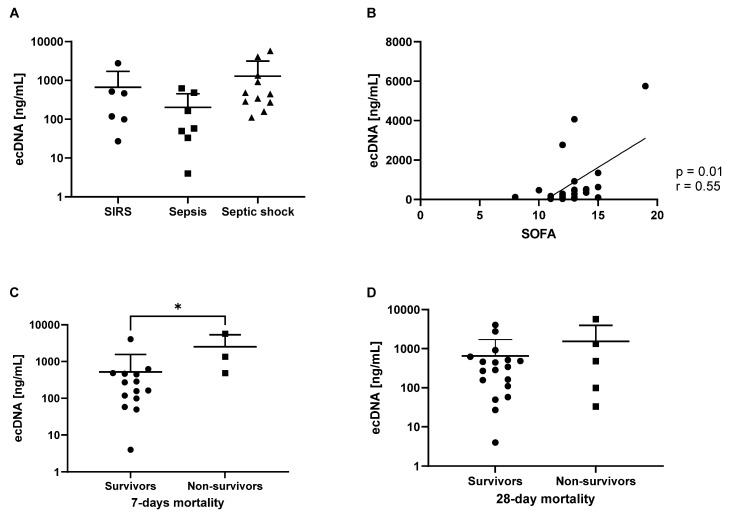
Total plasma ecDNA concentrations in patients diagnosed with SIRS, sepsis, and septic shock (**A**). The correlation of total ecDNA and SOFA score (*p* = 0.01; r = 0.55) in all patients (**B**). The comparison of total ecDNA in sepsis survivors and non-survivors (*p* = 0.04) by day 7 (**C**). The comparison of total ecDNA in sepsis survivors and non-survivors by day 28 (**D**)—means + standard deviations are shown on a logarithmic scale. *—*p*<0.05.

We divided 27 patients into groups based on the diagnosis received at admission. Of these patients, 8 were diagnosed with systemic inflammatory response syndrome (SIRS), 7 were diagnosed with sepsis, and 12 were diagnosed with septic shock, as shown in Table 2. No significant differences were found in terms of plasma ecDNA concentrations between patients with SIRS, sepsis, and septic shock (Figure 1A). We demonstrated moderately positive correlations (Figure 1B) between ecDNA concentrations and the clinical SOFA score (r = 0.55; *p* = 0.01). To assess whether ecDNA had a prognostic value, we further divided patients into survivors (20 patients; 14 men and 6 women) and non-survivors (7 patients; 3 men and 4 women) and compared total ecDNA concentrations in patients who survived the first 7 days and 28 days after ICU admission. The ecDNA concentrations were higher by 485% in non-survivors than in survivors (*p* = 0.04) by day 7 (Figure 1C). No significant differences were found in ecDNA between survivors and non-survivors by day 28 (Figure 1D). The average SOFA score of non-survivors was 15 by day 7 and 13 by day 28, while the average SOFA score of survivors was on average 12 by days 7 and 28.

### 3.2. Subcellular Origin of ecDNA Does Not Differ in Patients with SIRS, Sepsis and Septic Shock

**Figure 2:**
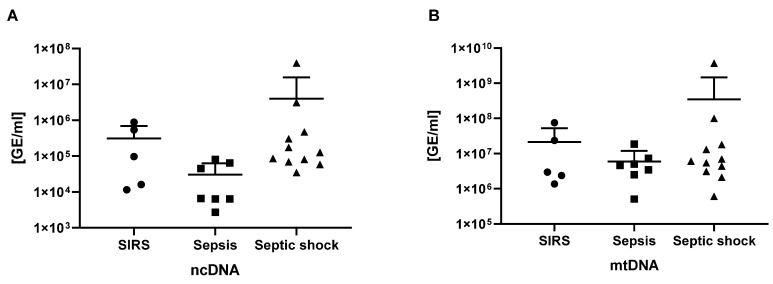
Nuclear DNA (ncDNA) (**A**) and mitochondrial DNA (mtDNA) in patients diagnosed with SIRS, sepsis, and septic shock (**B**)—means + standard deviations are shown on a logarithmic scale. GE—genome equivalents.

We hypothesized that ncDNA and mtDNA would be higher in patients with sepsis and septic shock with worse prognoses. The subcellular origin of ecDNA was analyzed using quantitative real-time PCR as well as primers for nuclear (beta-globin) and mitochondrial (D-loop) DNA. No significant differences were found between the groups in ncDNA (Figure 2A) and mtDNA (Figure 2B).

### 3.3. DNase Activity Was Similar in Patients with SIRS, Sepsis and Septic Shock

**Figure 3:**
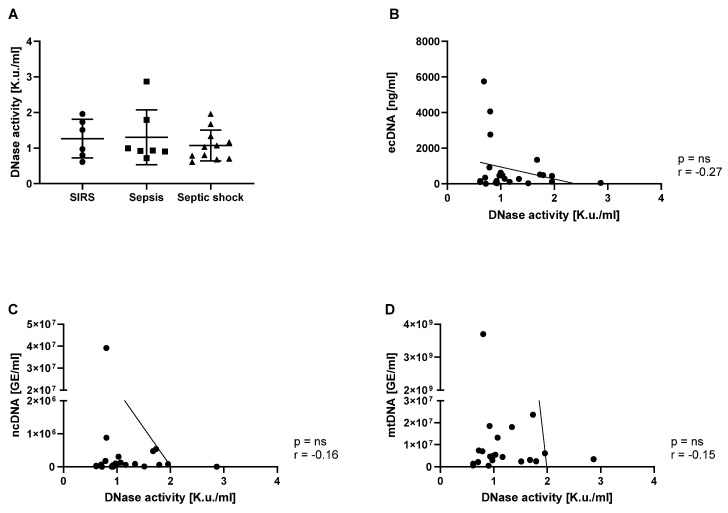
DNase activity in patients diagnosed with SIRS, sepsis, and septic shock (**A**). The correlation of total ecDNA and DNase activity (*p* = ns; r = −0.27) in all patients (**B**). The correlation of ncDNA and DNase activity (*p* = ns; r = −0.16) in all patients (**C**). The correlation of mtDNA and DNase activity (*p* = ns; r = −0.15) in all patients (**D**).

We hypothesized that there would be a lower DNase activity in patients with sepsis. Based on our analyses, DNase activity was similar in all groups of patients (Figure 3A). A weak, negative, and non-significant correlation (r = −0.27; *p* = ns) was found between total DNA concentration and DNase activity in these patients (Figure 3B). Similarly, we did not find associations between DNase activity and ncDNA (r = −0.16; *p* = ns) or mtDNA (r = −0.15; *p* = ns) in plasma (Figure 3C,D). 

## 4. Discussion

In this study, we analyzed ecDNA as a potential prognostic biomarker in patients admitted to ICU after being diagnosed with SIRS, sepsis, or septic shock. While we did not observe differences in ecDNA concentrations in patients grouped based on their diagnosis, we demonstrated moderately positive correlations (r = 0.55; *p* = 0.01) between ecDNA and the SOFA score. Our finding suggests that patients with worse clinical manifestation of sepsis have higher ecDNA levels at admission. Next, we decided to analyze patients based on their survival. We showed that the ecDNA concentration being higher in non-survivors than in survivors (*p* = 0.04) predicted death by day 7, but not by day 28. A higher mortality by day 7 has been shown for patients with higher ecDNA before [13]. Our results are in line with a recent meta-analysis showing the prognostic value of ecDNA for sepsis with regard to sensitivity and specificity [19]. However, the meta-analysis and most of the included studies ignored the subcellular origin of ecDNA.

A recent study analyzed ncDNA and mtDNA in plasma using quantitative PCR, similarly to our study. They obtained a similar outcome, showing the good diagnostic characteristics of ncDNA in combination with the SOFA score [20]. Our study also included the total plasma ecDNA, which might be more relevant due to the limitations of PCR with regard to the length of detected fragments. It is noteworthy that oxidized ecDNA and DNA fragments, binding through TLR9, could possibly serve as stress signals and exacerbate inflammation [21]. Unfortunately, we have not analyzed further the biochemical modifications of ecDNA that could be of immunological relevance. However, we have analyzed the DNase activity that could modulate the turnover and, thus, the pro-inflammatory characteristics of ecDNA.

It is known that neutrophils play an important role in the killing of bacteria by producing neutrophil extracellular traps (NETs) [22]. The scaffold of NETs is made from DNA and NETs contribute to the ecDNA pool in plasma, especially in inflammatory diseases, but also in cancer and, interestingly, also after physical exercise [23,24,25]. Unfortunately, we have not analyzed the other components of NETs and, so, we can only speculate that NET production in sepsis contributes to the ecDNA pool in our study. On the other hand, the available ELISA tests for NETs are error-prone and difficult to interpret [26].

MtDNA has been analyzed using a standard protocol that includes a second round of high-speed centrifugation. It has been shown that most of the extracellular mtDNA in plasma is large enough to be pelleted and, thus, discarded in this way [27]. How this affects the observed associations is not clear, and future studies should include the analysis of microparticle-associated ecDNA [28]. The form of mtDNA could also affect resistance against nucleases, as histones that protect ncDNA do not protect mtDNA.

DNase I activity is important for cleaving ecDNA in blood plasma [16]. Additionally, the deficiency of DNase I and DNase1L3 increased susceptibility to bloodstream infection, suggesting the importance of both enzymes during the control of bacterial infection [29] and NET degradation [30]. According to our data, the ecDNA concentration being higher in non-survivors predicts death by day 7. Thus, we expected lower DNase activity in these patients. In our study, the compared groups of patients (SIRS, sepsis, septic shock) did not differ in terms of DNase activity measured, using a DNA-containing gel. This method is not ideal, especially since the substrate for the enzyme consists of purified DNA and not chromatin or nucleosomes, as it does in vivo.

Several experimental studies of murine sepsis have shown the positive effects of DNase treatment on sepsis outcome, even demonstrating decreased mortality in mice with sepsis [17,31]. In a direct comparison, the effect was similar to the treatment with heparin [32]. A very interesting finding was that the application of DNase I too early in the course of sepsis might have a negative effect, potentially due to the interference with the antimicrobial activity of ecDNA/NETs [31]. Dual-acting DNase, combining the activities of DNase 1 and 1L3, can be used for the cleavage of ecDNA and NETs [33]. Sepsis seems to increase endogenous DNase activity [34], but the role of endogenous DNase activity in sepsis has not been studied in detail yet. However, it has been hypothesized that a high level of DNase activity could be protective [35]. The measurement of DNase activity using the SRED method is a limitation of our study; specifically, measuring the activity of each enzyme is needed. At this point, however, we were not able to establish a specific method for measuring the DNase 1l3 activity, for example, that could of relevance for this study.

Despite the relatively low number of patients, our findings indicate that ecDNA could serve as a valuable biomarker in terms of predicting sepsis outcomes. Interestingly, neither mtDNA nor ncDNA, which had been quantified using PCR, had similar informative value. Similarly, DNase activity showed no association with the diagnosis or with the survival of patients. The low sample size does not allow testing for non-linear relationships. Another limitation of this study is its inability to correlate ecDNA with additional biochemical parameters due to limited access to the health records. To establish the role of ecDNA as a prognostic biomarker, further investigations within a larger patient cohort and the analysis of biochemical parameters and microparticle-associated and extracellular vesicle-associated ecDNA is needed.

## Figures and Tables

**Table 1:** All patients’ health and demographic parameters.

Patient ID	Sex	Age	Height (m)	Weight (kg)	BMI	SOFA
1	M	28	1.94	90	23.91	12
2	M	51	1.64	64	23.80	15
3	M	74	1.85	95	27.76	15
4	M	72	1.80	85	26.23	15
5	M	58	1.67	63	22.59	12
6	M	46	1.85	90	26.30	12
7	F	59	1.68	60	21.26	13
8	M	64	1.66	74	26.85	11
9	M	70	1.80	95	29.32	13
10	F	81	1.62	65	24.77	8
11	F	70	1.75	72	23.51	13
12	F	68	1.60	85	33.20	19
13	F	84	1.60	55	21.48	10
14	F	50	1.58	50	20.03	12
15	M	61	1.95	95	24.98	13
16	M	59	1.80	120	37.04	13
17	M	57	1.70	70	24.22	14
18	F	55	1.58	60	24.03	11
19	M	59	1.73	86	28.73	13
20	M	68	1.75	85	27.76	12
21	F	73	1.68	50	17.72	14
22	M	42	1.80	85	26.23	13
23	M	50	1.80	85	26.23	11
24	F	45	1.65	60	22.04	14
25	1	70	1.69	75	26.26	15
26	0	55	1.70	80	27.68	9
27	0	49	1.85	75	21.91	13

Legend: M—men; F—women; BMI—Body Mass Index; SOFA—sequential organ failure assessment score.

**Table 2:** All patients’ group divisions: organized based on diagnosis.

Diagnosis	SIRS	Sepsis	Septic Shock
**Number of patients**	8	7	12
**Sex (men/women)**	4/4	5/2	8/4
**Average age**	58	63	59
**SOFA score (min/max)**	8/15	11/15	12/19

## Data Availability

The raw data supporting the conclusions of this article are available from the authors upon reasonable request.
